# Decreased activity of piriform cortex and orbitofrontal hyperactivation in Usher Syndrome, a human disorder of ciliary dysfunction

**DOI:** 10.1007/s11682-021-00594-6

**Published:** 2021-11-30

**Authors:** Sónia Ferreira, Isabel Catarina Duarte, André Paula, Andreia C. Pereira, João Carlos Ribeiro, Hugo Quental, Aldina Reis, Eduardo Duarte Silva, Miguel Castelo-Branco

**Affiliations:** 1 Institute for Biomedical Imaging and Translational Research (CIBIT), Faculty of Medicine, University of Coimbra, 3000-548 Coimbra, Portugal; 2 Institute of Nuclear Sciences Applied to Health (ICNAS, CIBIT), University of Coimbra, 3000-548 Coimbra, Portugal; 3 Otolaryngology, Centro Hospitalar e Universitário de Coimbra (CHUC), 3000-075 Coimbra, Portugal; 4 Ophthalmology Unit, Centro Hospitalar e Universitário de Coimbra (CHUC), 3000-075 Coimbra, Portugal

**Keywords:** Odor discrimination, Olfaction, Piriform, Orbitofrontal cortex, Functional Magnetic Resonance Imaging (fMRI)

## Abstract

Usher syndrome (USH) is a condition characterized by ciliary dysfunction leading to retinal degeneration and hearing/vestibular loss. Putative olfactory deficits in humans have been documented at the psychophysical level and remain to be proven at the neurophysiological level. Thus, we aimed to study USH olfactory impairment using functional magnetic resonance imaging. We analyzed differences in whole-brain responses between 27 USH patients and 26 healthy participants during an olfactory detection task with a bimodal odorant (*n*-butanol). The main research question was whether between-group differences could be identified using a conservative whole-brain approach and in a ROI-based approach in key olfactory brain regions. Results indicated higher olfactory thresholds in USH patients, thereby confirming the hypothesis of reduced olfactory acuity. Importantly, we found decreased BOLD activity for USH patients in response to odorant stimulation in the right piriform cortex, while right orbitofrontal cortex showed increased activity. We also found decreased activity in other higher-level regions in a whole brain approach. We suggest that the hyper activation in the orbitofrontal cortex possibly occurs as a compensatory mechanism after the under-recruitment of the piriform cortex. This study suggests that olfactory deficits in USH can be objectively assessed using functional neuroimaging which reveals differential patterns of activity both in low- and high-level regions of the olfactory network.

**Supplementary Information:**

The online version contains supplementary material available at 10.1007/s11682-021-00594-6.

## Highlights

1. Psychophysical olfactory deficits are present in Usher Syndrome, a ciliary disorder.

2. USH patients show decreased BOLD activity in the right piriform olfactory cortex;

3. USH patients show increased activity in the orbitofrontal olfactory cortex;

4. USH patients show patterns of decreased activity in high-level cortical regions;

5. Functional neuroimaging unravels USH olfactory deficits at the population level.

**Supplementary Information:**

The online version contains supplementary material available at 10.1007/s11682-021-00594-6.

## Introduction

Usher syndrome (USH) is a rare and heterogeneous group of diseases both from the genotypic and phenotypic point of view. It is characterized by retinitis pigmentosa and sensorineural hearing loss (Mathur & Yang, 2015; Toms et al., 2015). USH represents the most frequent cause of deaf-blindness and is inherited in an autosomal recessive pattern (Mathur & Yang, 2015; Toms et al., 2015). This condition is classified into three clinical types. Type I USH (USH1) patients suffer from severe to profound sensorineural congenital deafness, vestibular areflexia, and early onset retinitis pigmentosa. Type II USH (USH2) is characterized by mild to severe hearing loss, the absence of vestibular areflexia, and a later onset of retinitis pigmentosa. Type III USH (USH3) is the rarest type of the disease featuring progressive hearing loss, and variable vestibular dysfunction and time of onset of retinitis pigmentosa (Bonnet & El-Amraoui, 2012; Millán et al., 2011). The pathogenesis of USH has been associated with overall ciliary dysfunction, and thus, this disease has been described as a sensory ciliopathy (Mathur & Yang, 2015; Toms et al., 2015). Indeed, cells in the inner ear and the photoreceptor cells of the retina are known to be affected in USH (Millán et al., 2011) and have been linked to cilia defects (Toms et al., 2015). Since olfactory receptor cells are ciliated, the hypothesis of olfactory loss in USH has emerged (Ribeiro et al., 2016). However, olfactory function in USH patients has been poorly studied and results are mixed. Previous behavioral reports identified evidence for olfactory dysfunction in USH patients during odorant identification and detection tests (Giménez Vaillo et al., 1991; Zrada et al., 1996). Studies of nasal mucosa also suggested that ciliary cells might be defective (Arden & Fox, 1979; Marietta et al., 1997). Other authors found a genetic linkage between USH1 and the human olfactory marker protein, present in olfactory neurons of the olfactory bulb epithelium (Evans et al., 1993). Moreover psychophysical assessment reveals consistent olfactory loss in USH (Ribeiro et al., 2016). A study on brain structural integrity also showed that the olfactory sulcus was shallower in USH patients when compared with the control group (Ramos et al., 2019). Studies in mice have also provided evidence for USH olfactory impairment (Jansen et al., 2016; Sahly et al., 1997; Wolfrum et al., 1998). Nonetheless, other authors did not report reduced olfactory capacities in USH patients concerning the differential responses to odorant-evoked hedonic sensations, olfactory discrimination threshold, and odor identification (Marietta et al., 1997; Seeliger et al., 1999; Steiner & Abraham, 1978). In summary, olfactory deficits are often encountered in USH patients and olfactory testing may be important to help to distinguish among different USH forms (Ribeiro et al., 2016). However, the olfactory deficit in USH patients has been mainly evaluated by psychophysical tests and not by imaging methods.

Olfactory information is received by the first-order neurons in the nasal olfactory mucosa which projects to the second-order neurons of the olfactory bulb via the olfactory nerve. The olfactory bulb projects, via the lateral olfactory tract, to the piriform cortex, amygdala, and rostral entorhinal cortex. Then, these regions connect to higher-order brain areas: the orbitofrontal cortex (Rolls, 2015), cingulate cortex, insula, thalamus, hypothalamus, and hippocampus (Brand et al., 2001; Gottfried, 2006; Savic, 2005, 2002, 2001; Savic et al., 2000; Seubert et al., 2013a). Decreased activation levels in the central olfactory system assessed by functional magnetic resonance imaging (fMRI) have been linked to olfactory dysfunction in hyposmia and anosmia. Areas with reduced activation were mainly localized in the piriform cortex, amygdala, orbitofrontal cortex, cingulate gyrus, hippocampus, and insula (Frasnelli & Hummel, 2007; Henkin & Levy, 2002; Iannilli et al., 2011, 2007). However, this has not yet been addressed in USH, which provides a new genetically determined model to study to neural impact of olfactory dysfunction.

We aimed to study olfactory impairment in USH using fMRI in addition to the measurement of olfactory detection thresholds. We intended to determine which cortical regions, in the core and extended olfactory processing network, are associated with olfactory loss in USH patients. We used both exploratory (whole brain) and hypothesis driven approaches focused on olfactory regions. In the exploratory approach on the data, we first analyzed group differences in brain responses during an odor detection task between USH patients and age- and sex-matched healthy participants (controls). To focus on core olfactory regions, we then compared groups for supra versus infra threshold odor levels. Our hypothesis is that USH patients have decreased olfactory performance (higher olfactory detection thresholds) and weaker responses in brain regions, in particular the piriform cortex, associated with core olfactory processing.

## Material and methods

### Participants

The study was conducted in accordance with the Declaration of Helsinki and was approved by the Ethics Committee of Faculty of Medicine of University of Coimbra, Portugal. Written informed consent was obtained from all participants.

Sixty-six participants were recruited, however 13 were excluded as described next. A structured medical history review was conducted to exclude visual, olfactory, and auditory alterations in control participants (2 controls were excluded). A full ENT (Ear Nose Throat) clinical evaluation comprising nasal endoscopy and a rhinology examination was also performed to all participants by an experienced physician (JCR) to exclude pathologies potentially causing olfactory dysfunction (e.g. post-traumatic olfactory dysfunction, sinonasal disease, malignant tumor, recent radiotherapy or chemotherapy, or post-upper respiratory tract infection) (1 control was excluded) (Ribeiro et al., 2016). Additional exclusion criteria for all participants included any intracranial abnormality identified on the MRI images as accessed by a neuroradiologist and incorrect task execution during fMRI acquisitions (8 patients and 2 controls were excluded). Montreal Cognitive Assessment test (MoCA) was used in USH patients to exclude dementia putatively causing olfactory impairment (Freitas et al., 2011).

Fifty-three participants were included in the final analysis. Twenty-seven USH individuals (19 male and 8 female; age range from 32 to 78 years; age 44.00 (14.00) years [median (interquartile range)]; 25 right-handed and 2 left-handed; 4 USH1, 21 USH2, and 2 USH3) and 26 control subjects (18 male and 8 female; age range from 32 to 74 years; age 42.00 (16.30) years; 25 right-handed and 1 left-handed) were included in the study. Both groups were matched for age [*U* = 381.00, *p* = 0.593], sex [χ^2^_(1)_ = 0.01, *p* = 0.582], and handedness [χ^2^_(1)_ = 0.31, *p* = 0.514] ratios. Patients were recruited in collaboration with the Otorhinolaryngology Unit at *Centro Hospitalar e Universitário de Coimbra*, Portugal. USH patients were diagnosed using clinical ophthalmological and otorhinolaryngological criteria by two experienced physicians (EDS and JCR, respectively) (Smith et al., 1994). The diagnosis was later confirmed by genetic testing. The control group were healthy volunteers recruited locally.

### Psychophysical testing of olfactory thresholds

The olfactory threshold corresponds to the lowest concentration of an odorant molecule that can be detected by an individual (Braun et al., 2014). The olfactory threshold test was executed as a single staircase procedure with a set of 8 solutions of *n*-butanol with a concentration ranging from 4% to 0.002% following a 1/3 dilution with water as a solvent (Croy et al., 2009). The test was done birhinally and the butanol odorant was presented to the participants using 250 ml bottles with 60 ml butanol solution. Butanol is an odorant that activates the olfactory/trigeminal systems (Iannilli et al., 2007; Vedaei et al., 2013).

### Brain imaging procedures

Scanning was performed on a 3 T scanner (Magneton TrioTim, Siemens AG, Germany) at the Portuguese Brain Imaging Network, using a 12-channel birdcage head coil. Two T1-weighted Magnetization-Prepared Rapid Acquisition with Gradient Echo (MPRAGE) sequences, with 1×1×1 mm^3^ voxel size, Repetition Time (TR) 2.53 s, Echo Time (TE) 3.42 ms, Flip Angle (FA) 7°, Field Of View (FOV) 256×256 mm^2^, and 176 slices were acquired from each participant. The functional sequences consisted in single shot Echo Planar Imaging (EPI) acquired 30 deg in the axial plane orthogonal to the Anterior Commissure–Posterior Commissure plane (AC-PC) covering the whole brain, with 3×3×3 mm^3^ voxel size, TR 3 s, TE 30 ms, FA 90°, FOV 256×256 mm^2^, 43 slices, and 86×86 imaging matrix. This slice orientation was chosen to minimize the signal dropout in the orbitofrontal and medial temporal areas caused by susceptibility artifacts (Tabert et al., 2007). Prior to each functional sequence, a multi-echo EPI was also acquired to correct for EPI distortion due to susceptibility artifacts, with 3.7×3.7×3.0 mm^3^ voxel size, TR 0.4 s, TE_1_ 4.92 ms, TE_2_ 7.38 ms, FA 60 deg, FOV 235×235 mm^2^, 36 slices, and 64×64 imaging matrix.

#### Olfactory task

**Fig. 1:**
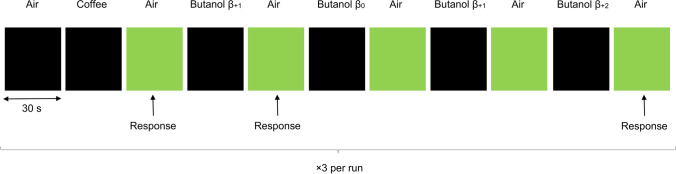
Illustration of part of the olfactory task performed inside the magnetic resonance imaging scanner. Four concentrations of *n*-butanol were presented in a random staircase design with 4 levels: the butanol threshold concentration (β_0_) determined before entering the scanner, one concentration below (β_-1_), one concentration above (β_+1_), and one concentration further above (β_+2_). Starting at a random concentration (in the figure, β_+1_), participants were instructed to breathe normally during odorant presentation blocks (black screen) and to press a button whenever they detected the odorant after the green screen appeared. Each time the participant detected the odorant, the next concentration was lower, otherwise, it was higher. Odorless air was used as a 0 % control condition and supra-threshold coffee odorant was also released to prevent odorant saturation. For explanatory purposes, we showed a case where the random concentration at the first butanol block was β_+1_ (detected), followed by a concentration decrease to β_0_ (not detected), followed by a concentration increase to β_+1_ (not detected), and followed by a concentration increase to β_+2_ (detected)

Four concentrations of butanol were presented in a random staircase design with the following levels: the butanol threshold concentration (β_0_) determined before entering the scanner, one concentration below (β_-1_), one concentration above (β_+1_), and one concentration further above (β_+2_) – Fig. 1. Participants were instructed to breathe normally and to smell without sniffing (Vedaei et al., 2013) during the odorant presentation blocks (black screen), and to press a button whenever they detected the odorant after a green or white (for patients with a severe visual loss) screen appeared. Odorant presentation started at a random concentration. Whenever the participant detected the odorant, the next concentration was lower, otherwise, it was higher to ensure an adaptive staircase design. Participants were not specifically told that the concentrations varied, however we told them they could occasionally not sense anything. Odorless air was used as a 0 % control condition and supra-threshold coffee odorant was also released three times per run to prevent odorant saturation. Odorant release blocks (black screen) were designed to give enough time for detection due to the putative olfactory impairment in patients (Fig. 1) (Tabert et al., 2007). Each run lasted 16 min and 30 s, with 3 blocks of coffee, 12 blocks of butanol, and 18 blocks of odorless air. Two functional runs were acquired per participant within the same session, except for 4 patients who asked to leave the scanner after the first run due to fatigue.

The stimulus was designed using Matlab 2010b (The MathWorks, Inc., USA) with Psychophysics Toolbox 3 extensions. The coloured screen was presented in a back-projection Liquid Crystal Display monitor (NordicNeuroLab, Norway) with a mirror mounted above the coil. Responses were collected with an fMRI response pad (Lumina LU400-PAIR, Cedrus Corporation, USA). The odorants were presented using an olfactometer (Mag Design and Engineering, Redwood City, USA), which allowed the presentation of up to 6 different odorants and was controlled using the Matlab script. The olfactometer was kept in the control room while the individual ducts containing air passed to the MRI room through the waveguide and were connected to the individual odorant containers. The air flow was delivered simultaneously to both nostrils, through Teflon-tubing.

#### Image processing and analysis

Image processing and analysis were carried out using BrainVoyager QX 2.6.1 (Brain Innovation BV, The Netherlands). The two high-resolution anatomical images were averaged to improve the signal-to-noise ratio. Anatomical volumes were re-oriented in relation to AC-PC and transformed to Talairach (TAL) coordinate system. Anatomical images were used for the projection of cortical functional maps during the olfactory task.

Before preprocessing of the functional images, we used Anatabacus, a plugin for BrainVoyager software, following the standard procedure to correct for EPI geometric distortion due to susceptibility artifacts (Breman et al., 2009). Scan time correction, temporal high-pass filtering (2 cycles per run) and correction for small inter-scan head movements were applied during preprocessing. After spatial normalization, spatial smoothing (FWHM 6 mm) was applied. A General Linear Model (GLM) with 5 predictors (Coffee, Butanol β_-1_, Butanol β_0_, Butanol β_+1_, and Butanol β_+2_) was applied for each run from each participant. Additional predictors were considered to correct for within-run head movement. An habituation effect was described before for olfactory stimuli (Georgiopoulos et al., 2018; Poellinger et al., 2001; Sobel et al., 2000). However, as the present study includes a clinical group in which habituation effects might be distinct, we choose to have larger blocks. As demonstrated in supplementary figure 1, we still verified a strong habituation effect on the hemodynamic response with an undershoot occurring within the stimulation block, starting nearly at the middle of the block. Therefore, we accounted only for the first half part of the stimulation block. A multi-study GLM combining all participants’ predictors for each run was performed. We run a conservative approach and compared whole-brain activation between USH and control groups contrasting the response during the butanol blocks [β_-1_ (one concentration below the olfactory threshold), β_0_ (olfactory threshold concentration), β_+1_ (one concentration above the olfactory threshold), and β_+2_ (concentration two levels above the olfactory threshold)].

Regions of interest (ROIs) were defined in the orbitofrontal cortex and in the piriform cortex. Right and left orbitofrontal regions were defined selecting the peak voxel of the contrast above and defining a spheric ROI (257 mm^3^) centered on that coordinate. Coordinates are described in terms of center of gravity and localization is shown in the Results section. Using all butanol conditions versus baseline, the piriform cortex was defined using a within-subject approach of all participants, while for the orbitofrontal cortex a between-subject approach was used (USH vs Controls). To prevent circularity, the ROI-based approach was performed using the specific contrast β_+2_ vs β_-1_ (the most supra-threshold versus the infra-threshold conditions).

### Statistical analysis

Statistical analyses were performed with IBM SPSS Statistics 22 (IBM Corporation, USA). Normality assumption for all variables was tested using Shapiro-Wilk’s test. Parametric tests were used for normally distributed data. Otherwise, non-parametric tests were used. The significance level was set at *α* = 0.05. Bonferroni correction for multiple comparisons was applied (values presented as *corrected p*).

The USH group was mostly comprised of USH2 patients (n = 21) in comparison to USH1 (n = 4) and USH3 patients (n = 2). Thus, no statistical tests were performed between the different USH types.

## Results

### Psychophysical testing of olfactory threshold

**Fig. 2:**
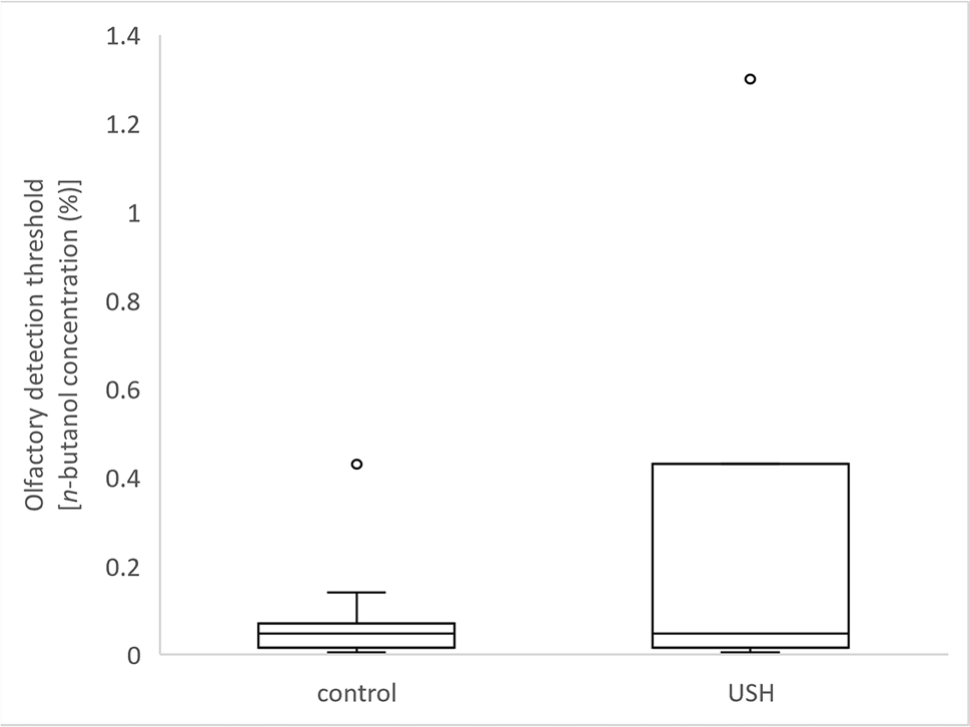
Representation of the different olfactory threshold (Mann-Whitney test *U* = 482.50, *p* = 0.016) between Usher (USH) and healthy participants measured by the psychophysical test. Olfactory detection thresholds were higher for USH patients than controls (decreased olfactory acuity). The graphs represent the Tukey’s boxplot of the data

The butanol threshold concentration was significantly different between USH patients and the control group [Mann-Whitney test *U* = 482.50, *p* = 0.016], such that thresholds were higher for USH [median (interquartile range) 0.047 (0.414) %] than controls [0.047 (0.054) %] – Fig. 2. This result indicates that USH patients have decreased olfactory capacity when compared to control subjects concerning olfactory detection.

### Behavioral detection responses to the olfactory task during fMRI scanning

Task response percentages (number of odorant detections/number of odorant presentations × 100) for each butanol concentration were not different between USH and control participants for the first and the second fMRI runs [Mann-Whitney test, corrected *p* ≥ 0.196]. This result was expected because the 4 butanol concentrations were adjusted according to each participant’s olfactory threshold. Moreover, there were no within-group differences between the 2 runs for each butanol concentration [Wilcoxon-signed rank test, corrected *p* ≥ 0.636], showing that behavioral response patterns remained stable across the whole experiment. There was a statistically significant effect of concentration within the USH group [Friedmann test, first run χ^2^_(3)_ = 29.65, *p* = 2.000×10^-6^ and second run χ^2^_(3)_ = 15.43, *p* = 0.001] and within the control group [first run χ^2^_(3)_ = 18.46, *p* = 3.530×10^-4^ and second run χ^2^_(3)_ = 11.69, *p* = 0.009] as expected due to the different butanol concentrations used during the task. Both groups responded to a larger extent to the 2 highest concentrations of butanol (above the threshold β_+1_ and β_+2_) when compared to the 2 lowest concentrations (the threshold β_0_ and below the threshold β_-1_). For controls, the detection response to β_+1_ was statistically different from the response to β_-1_ [*Z* = -3.16, corrected *p* = 0.012] and β_0_ [*Z* = -2.98, corrected *p* = 0.018] during the first run. For USH patients, the response to β_-1_ was statistically different from the responses to β_+1_ [*Z* = -3.46, corrected *p* = 0.006] for both runs, and from the concentration β_+2_ [*Z* = -3.88, corrected *p* = 0.001] during the first run.

### Brain responses during the olfactory task

**Fig. 3:**
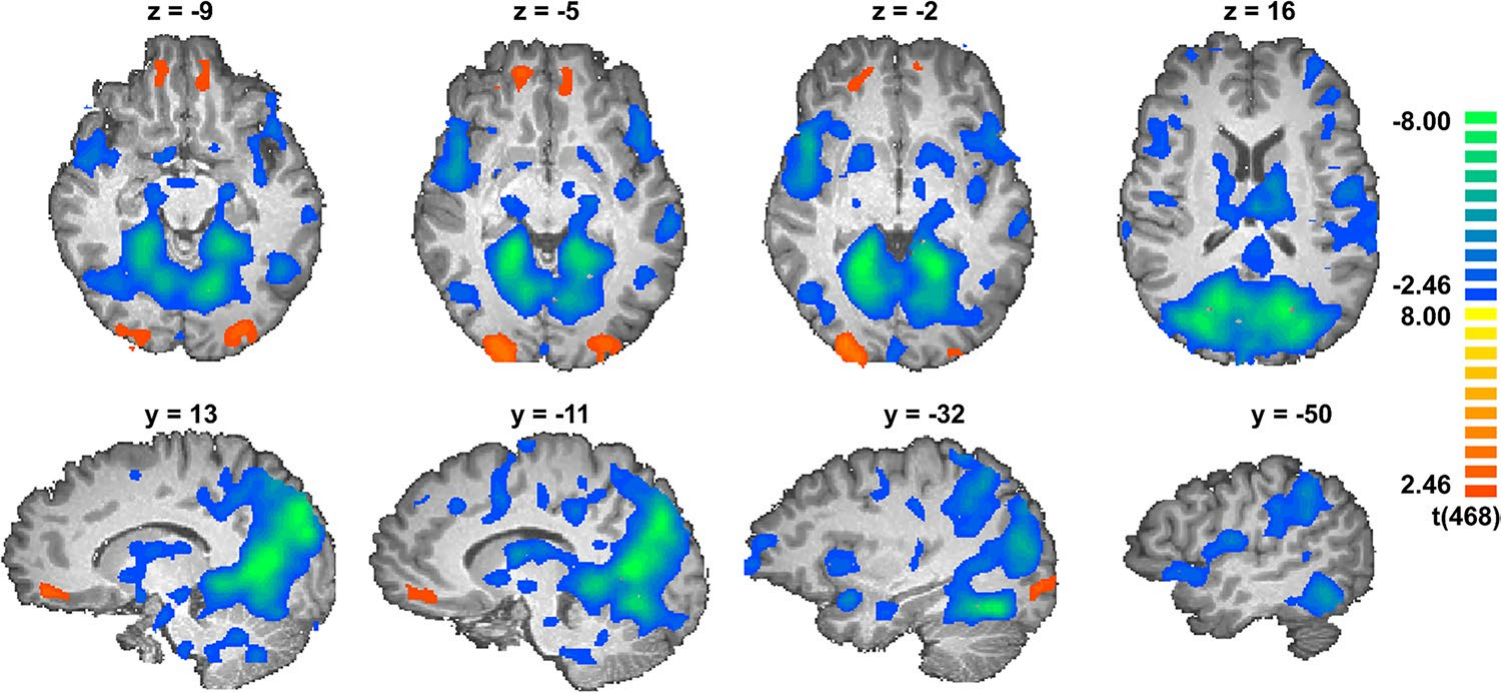
Statistical map showing significant differences between the Usher and Control groups (-2.46 > t > 2.46, p < 0.05, FDR corrected and minimum voxel size of 25 mm^3^). Regions were identified in a whole-brain analysis using all butanol concentrations [β_-1_ (one concentration below the olfactory threshold), β_0_ (the olfactory threshold), β_+1_ (one concentration above the olfactory threshold), and β_+2_ (concentration two levels above the olfactory threshold)] versus the 0 % control condition (odorless air). Left side on transversal slices corresponds to right hemisphere

We run a conservative approach and compared whole-brain activation between USH and control groups contrasting the response during the butanol blocks (β_-1_ + β_0_ + β_+1_ + β_+2_ vs odorless air). The analysis of all 4 butanol concentrations together elicited increased activity in the orbitofrontal and occipital cortex in the USH group when compared with the control group (-2.46 > *t* > 2.46, *p* < 0.05, FDR corrected and minimum voxel size of 25 mm^3^). The same contrast showed decreased activity bilaterally in the insula/operculum, ventral putamen, dorsal anterior cingulate and posterior cingulate, cuneus/precuneus/fusiform gyrus, frontal pole (Brodmann area [BA] 10), precentral gyrus (BA 6), and cerebellum in the USH group when compared with the control group. Details are described in Table 1 and presented in Fig. 3.

**Table 1:** Regions showing significant differences between Usher and Control group (-2.46 > *t* > 2.46, *p* < 0.05, FDR corrected and minimum voxel size of 25 mm^3^). Regions were identified in a whole-brain analysis using all butanol concentrations [β_-1_ (one concentration below the olfactory threshold), β_0_ (the olfactory threshold), β_+1_ (one concentration above the olfactory threshold), and β_+2_ (concentration two levels above the olfactory threshold)] versus the 0 % control condition (odorless air). H=hemisphere, R=right, L=left, BA=Brodmann area, OFC=orbitofrontal cortex, PFC=prefrontal cortex, ACC=anterior cingulate cortex

			peak			
region	H	x	y	z	t	p
OFC (BA 11, 12)	R	12	44	-8	3.40	0.000735
OFC (BA 11, 12)	L	-9	38	-8	3.09	0.002092
visual cortex (BA 18, 19)	R	27	-88	-2	4.47	0.00001
visual cortex (BA 18, 19)	L	-27	-85	-8	3.79	0.00017
insula/operculum	R	45	11	-2	-6.25	<0.000001
insula/operculum	L	-45	14	-8	-4.44	0.000011
ventral putamen	R	18	2	1	-4.46	0.00001
ventral putamen	L	-12	5	1	-3.79	0.000172
thalamus	R, L	-12	-7	13	-5.23	<0.000001
dorsal ACC/superior frontal gyrus (BA 24, 32, 6, 8)	R, L	3	-10	71	-4.70	0.000003
posterior cingulate	R, L	3	-31	40	-5.12	<0.000001
cuneus/precuneus/fusiform	R, L	-18	-64	7	-10.69	<0.000001
PFC (BA 10)	R	27	56	22	-3.65	0.000296
PFC (BA 10)	L	-30	59	4	-4.26	0.000024
middle frontal gyrus (BA 6)	R	36	5	25	-5.20	<0.000001
middle frontal gyrus (BA 6)	L	-42	-4	52	-3.75	0.000199
middle temporal gyrus	L	-63	-28	-5	-4.38	0.000015
cerebellum	R,L	9	-49	-29	-4.38	0.000014
brainstem	R,L	12	-22	-35	-4.19	0.000034

**Fig. 4:**
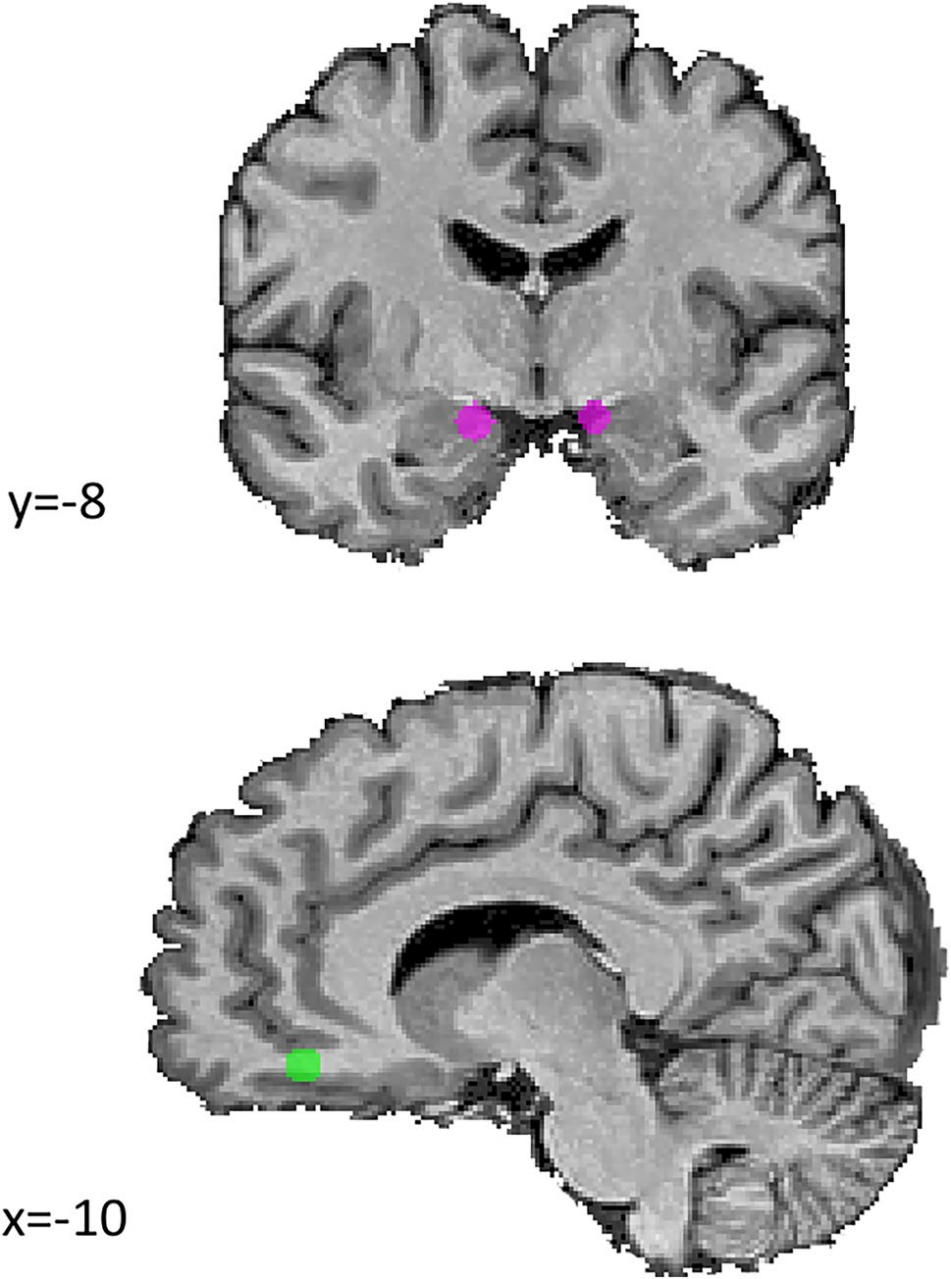
Regions selected for ROI based analysis. Bilateral spherical ROIs were defined in the orbitofrontal cortex (green) and in the piriform cortex (pink). These areas were used to test the contrast USH vs Controls considering the specific contrast β+2 vs β-1 (the most supra-threshold versus the infra-threshold conditions). Left side on coronal slice corresponds to right hemisphere

We further compare the groups in a ROI based approach, focusing on the olfactory processing areas: the orbitofrontal and the piriform cortices. To focus on odor processing (and cancel other non-olfactory task-related activations), we contrasted the most supra threshold condition with the concentration below the olfactory threshold (β_+2_ vs β_-1_) between groups. We found decreased activity in the right piriform cortex (F(1,51)=10.736, p=0.0019) and increased activity in the right orbitofrontal cortex (F(1,51)=6.168, p=0.016) by the USH groups when compared with the control group. The contralateral regions in the left hemisphere did not evidence statistically significant differences between groups. Details are described in Table 2 and ROIs are presented in Fig. 4.

**Table 2:** Region of interest analysis. Spherical ROIs are centered at the coordinate described. F-values and p-values are referred to the contrast USH vs Controls considering the contrast β_+2_ vs β_-1_ (the most supra-threshold versus the infra-threshold conditions) H=hemisphere, R=right, L=left, OFC=orbitofrontal cortex

		Center of gravity						
region	H	x	y	z	USH mean	Controls mean	F	p
OFC	R	12	44	-8	0.196	-0.111	6.168	**0.0163**
OFC	L	-9	38	-8	0.044	-0.084	1.907	0.1733
Piriform cortex	R	13	-8	-12	-0.212	0.134	10.736	**0.0019**
Piriform cortex	L	-12	-6	-11	-0.106	0.059	2.845	0.0977

## Discussion

We aimed to study olfactory impairment in Usher Syndrome across distinct processing levels using functional magnetic resonance imaging. We used a conservative whole-brain approach to identify response changes in olfactory and high-level regions during an olfactory detection task between USH patients and age- and sex-matched healthy participants. In a hypothesis-driven ROI-based approach, we further investigated the group differences in the piriform cortex and in the orbitofrontal cortex, using an olfactory specific contrast. We found evidence for both psychophysical and distinct patterns of brain responses in core olfactory regions and also, as observed in the exploratory whole brain analysis, in regions beyond the olfactory circuitry.

The olfactory detection threshold was significantly increased in USH patients when compared to the control group, confirming our hypothesis of decreased olfactory performance in patients which is in line with previous studies (Giménez Vaillo et al., 1991; Ribeiro et al., 2016; Zrada et al., 1996).

In our fMRI experiment, we accounted for the individual detection threshold to tailor the staircase procedure to each participant. This was done to ensure that task difficulty and attentional demands were similar between USH and control groups, which was further confirmed by behavioral data analysis showing no differences between groups’ performance during the fMRI task.

The contrast of butanol odorant versus odorless air during the olfactory detection task elicited different activation patterns between groups in several brain regions. Specifically, USH patients showed significantly increased activity in the orbitofrontal cortex when compared to the healthy controls. This is remarkable because this region receives input from piriform cortex (which exhibited reduced activity in USH patients when compared with the control group in the ROI-based analysis). These regions were bilaterally localized and included the prefrontal cortex, insula, ventral putamen, superior frontal gyrus and middle frontal gyrus. Bimodal odorants (i.e. with both olfactory and trigeminal components), such as *n*-butanol, have been shown to produce bilateral brain activation (Albrecht et al., 2010). This is supported by the contralateral projection of the trigeminal nerve in the brain, in contrast to the ipsilateral projection of the olfactory nerve (Brand et al., 2001; Gottfried, 2006).

The insula has a crucial role in olfactory processing and activates under basic odor perception as well as higher order tasks (e.g. olfactory recognition and discrimination) (Frasnelli et al., 2010; Savic, 2005, 2001; Savic et al., 2000; Seubert et al., 2013a). This region integrates olfactory and trigeminal information and is involved in the processing of nociceptive information (Albrecht et al., 2010; Frasnelli & Hummel, 2007; Hummel et al., 2005; Lombion et al., 2009; Seubert et al., 2013a). Moreover, the insula/claustrum regions receive direct projections from the olfactory system (the piriform cortex and the amygdala) (Poellinger et al., 2001; Seubert et al., 2013a; Zald & Pardo, 2000). Previous studies reported less activation in the left insula during trigeminal stimulation in anosmic patients (Frasnelli & Hummel, 2007; Iannilli et al., 2007).

We also found altered activity in regions beyond the core olfactory circuitry, which is expected for this contrast as other task-related activity may appear beyond the olfactory processing itself. Olfactory processing is quite distinct from the topographical organization of other sensory modalities and it is quite segregated in widespread subcortical and cortical regions (Fjaeldstad et al., 2017). The superior frontal gyrus is involved in odor familiarity judgments, trigeminal stimulation, and odorant intensity discrimination (Albrecht et al., 2010; Brand et al., 2001; Frasnelli & Hummel, 2007; Hummel et al., 2005; Lombion et al., 2009; Savic, 2005; Seubert et al., 2013a; Vedaei et al., 2013). Gray matter volume loss was found in the superior frontal gyrus of anosmic patients (Bitter, Gudziol, et al., 2010b) and decreased white matter volume was found near the middle frontal gyrus in hyposmic patients (Bitter et al., 2010a). The putamen is interconnected with the amygdala and is involved in conscious odor processing during detection tasks (Seubert et al., 2013a). Thus, our results point to an overall functional reduction of high order circuits, in addition to core olfactory cortex.

Both in the whole brain and the ROI-based approaches, the USH group showed higher right OFC recruitment when compared with the control group. Opposite results were found in the right piriform cortex as identified in the ROI-based approach. Here, the USH group showed a reduced activity when compared to the control group.

The identified dissociation between piriform (which receives direct input from the olfactory bulb) and the orbitofrontal cortex (which is just upstream from the former regions) is intriguing. The piriform, entorhinal, cingulate and insular cortices are part of the main circuit for olfactory processing (Brand et al., 2001; Gottfried, 2006; Savic, 2005, 2002, 2001; Savic et al., 2000; Seubert et al., 2013a). On the other side, the orbitofrontal cortex is the main area receiving projections from the piriform and entorhinal regions. We found remarkable that patients showed higher activation in the orbitofrontal cortex, suggesting that the association between identification and stimulus valuation is enhanced in patients, possibly in a compensatory manner. Previous works reported that hyposmia and anosmia lead to reduced activation in the piriform cortex and cingulate gyrus (Frasnelli & Hummel, 2007; Henkin & Levy, 2002; Iannilli et al., 2011, 2007). Larger orbitofrontal volumes are associated with a greater flexibility for adaptation to odors (e.g. tasks involving perceptual decision-making, confidence judgment, perceptual learning, valence judgment, and multisensorial integration) (Seubert et al., 2013b). Previous work demonstrated that orbitofrontal activity increased after one week of olfactory deprivation in humans to optimize olfactory perception, whereas piriform responses decreased (Wu et al., 2012). Orbitofrontal cortex recalibrates to detect odors at lower concentrations (Wu et al., 2012). Thus, the orbitofrontal cortex in USH patients might be compensating the reduced input from other odor processing regions, such as the piriform cortex, by increasing its responsiveness - a putative form of functional plasticity, akin to other sensory domains.

## Conclusions

This study demonstrates olfactory sensory deficits in a disorder of ciliary dysfunction, Usher Syndrome, showed by changes in olfactory detection thresholds. This led to different activation patterns in piriform (reduced) and orbitofrontal (increased) regions of the core olfactory network in Usher syndrome. The dichotomic pattern of deactivation in the piriform, as compared to the activation in the orbitofrontal region, extends to other high-level cortical regions. The evidence that USH patients show decreased activation in right piriform and high-level regions and increased responses in the right orbitofrontal cortex is reminiscent of findings that are observed in other sensory systems. We suggest that this hyper activation in the orbitofrontal cortex possibly occurs in a compensatory manner, as it is the main area receiving projections from the piriform regions.

## Supplementary information

(PDF 475 kb)

## Abbreviations

USH: Usher Syndrome
USH1: Type I Usher Syndrome
USH2: Type II Usher Syndrome
USH3: Type III Usher Syndrome
OFC: Orbitofrontal cortex
fMRI: Functional Magnetic Resonance Imaging
