# Supplementary material for: Decreased activity of piriform cortex and orbitofrontal hyperactivation in Usher Syndrome, a human disorder of ciliary dysfunction
Source: Brain Imaging Behav. 2021 Nov 30;16(3):1176–85. doi: 10.1007/s11682-021-00594-6 (PMC9107447; doi:10.1007/s11682-021-00594-6)
Supplement: Supplementary file 1 — (PDF 475 kb) [file 11682_2021_594_MOESM1_ESM.pdf]

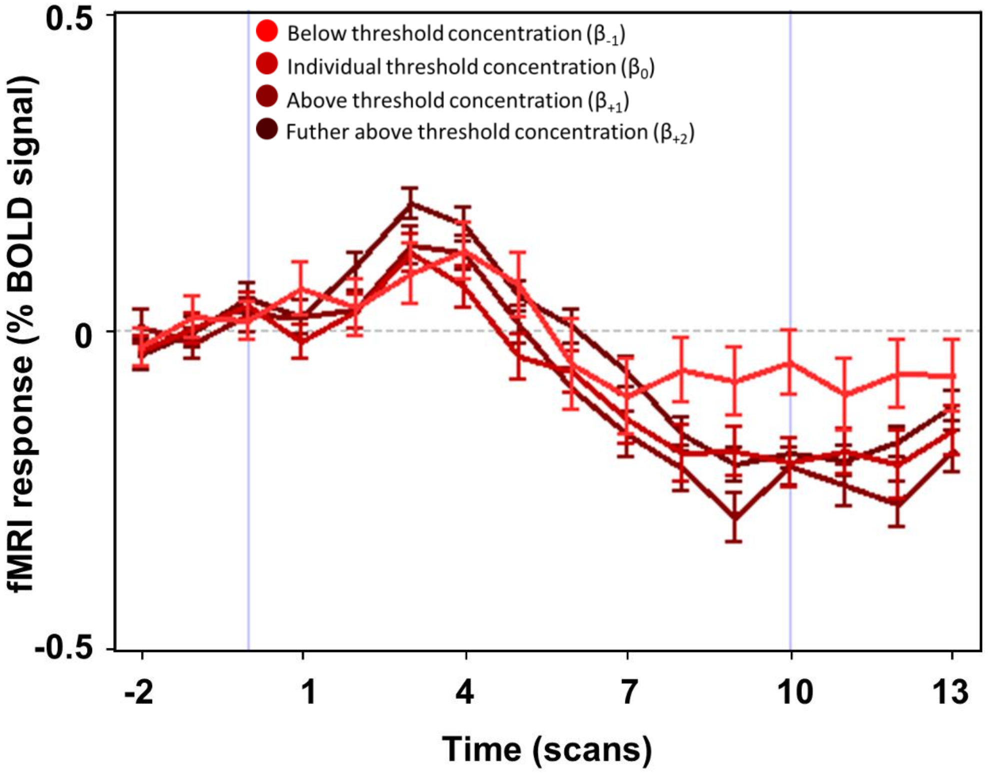

Supplementary Fig. 1 Percentage of BOLD signal changes during blocks of odor stimulation. The curves show a strong habituation effect on the hemodynamic response with an undershoot occurring within the stimulation block. One scan is 3 seconds. Darker the color red, more concentrated the butanol solution.
